# Strengthening social accountability in ways that build inclusion, institutionalization and scale: reflections on FHS experience

**DOI:** 10.1186/s12939-020-01341-x

**Published:** 2020-12-10

**Authors:** Sara Bennett, Eizabeth Ekirapa-Kiracho, Shehrin Shaila Mahmood, Ligia Paina, David H. Peters

**Affiliations:** 1 Department of International Health, Johns Hopkins Bloomberg School of Public Health, 614 N Wolfe St, Baltimore, MD USA; 2 Department of Health Policy, Planning and Management, Makerere University School of Public Health, P.O.Box 7072, Kampala, Uganda; 3 Health Systems and Population Studies Division, icddr,b, 68 Shaheed Tajuddin Ahmed Sarani, Mohakhali, Dhaka, 1212 Bangladesh

**Keywords:** Social accountability, Community score cards, Scale-up, Sustainability, Inclusion

## Abstract

This editorial provides an introduction to the special issue on “Lessons about intervening in accountability ecosystems: implementation of community scorecards in Bangladesh and Uganda”. We start by describing the rationale for this work in the two study countries. While our project, the Future Health Systems (FHS) project, had been working over the course of more than a decade to strengthen health services, particularly for low income households in rural areas, our teams increasingly recognized how difficult it would be to sustain service improvements without fundamental changes to local accountabilities. Accordingly, in the final phase of the project 2016–2018, we designed, implemented and assessed community scorecard initiatives, in both Bangladesh and Uganda, with the aim of informing the design of a scalable social accountability initiative that could fundamentally shift the dynamics of health system accountability in favor of the poor and marginalized.

We describe the particular characteristics of our approach to this task. Specifically we (i) conducted a mapping of accountabilities in each of the contexts so as to understand how our actions may interact with existing accountability mechanisms (ii) developed detailed theories of change that unpacked the mechanisms through which we anticipated the community scorecards would have effect, as well as how they would be institutionalized; and (iii) monitored closely the extent of inclusion and the equity effects of the scorecards. In summarizing this approach, we articulate the contributions made by different papers in this volume.

## Introduction

Over the past decade there has been a growing consensus that stronger accountability mechanisms are critical for improving health services and health [1–4]. While, on the one hand, there is evidence of the significant impact that strengthened accountability for health can have, there is also a substantive and growing body of evidence that documents the challenges involved in strengthening accountability, especially in terms of scaling up interventions [5, 6] integrating approaches into existing health systems [7–9] and ensuring inclusiveness [8].

In 2016, a UK Department for International Development-funded research consortium, Future Health Systems (FHS) had the opportunity to address this question of social accountability through our work program in Uganda and Bangladesh. The FHS consortium was launched in 2005, and had previously piloted community scorecards and balanced scorecards in Afghanistan [10, 11], and conducted research on community capabilities in Uganda, India, and Bangladesh [12]. Our prior work had demonstrated the potential for community scorecards to enhance community capacity to contribute to improved health services but also documented a number of challenges. These included ensuring the availability of skilled facilitators who could help to create a safe environment for participants to openly share views, and challenges related to sustaining the scorecards and integrating them into existing systems and structures [11]. Through their experience in working with communities, the FHS teams in Bangladesh and Uganda each identified a need to strengthen local level accountability and community engagement processes. For example, the Uganda team had until that point in the project focused on developing and implementing a package of interventions in Eastern Uganda to strengthen maternal, neonatal and child health services. However, the team recognized that to mobilize community resources and sustain activities beyond the initial incentive schemes, local level empowerment and accountability interventions were required. In Bangladesh, the FHS team intervened to improve access to qualified healthcare for the rural poor and increase the use of public sector health facilities in this connection. The team also recognized that without strengthening accountability for performance at the community level, the community-based programs that government was seeking to re-energize were going to be difficult to sustain. In both Uganda and Bangladesh it was hoped that strengthening local empowerment and social accountability would serve to reinforce the earlier interventions by addressing pernicious health system problems including poor attitudes of health workers, high absenteeism, neglect of duty, and misuse of resources.

Given the accumulating global literature about the potential power of social accountability approaches, combined with the views of country teams on the ground that an approach of this nature was necessary to secure hard fought gains, it seemed natural for the project to address this issue. However, as we developed a project strategy on social accountability, our team was very sensitive to a number of likely challenges.

First, we were very aware that any social accountability intervention would not be implemented in a vacuum, but would likely be layered upon multiple strata of power and accountability relations within the health system [13], as well as prior projects that had also sought to intervene in accountability relationships. Health systems are embedded within broader social structures, and typically reflect the prevailing power relations in society. While sometimes such structures might work to improve the health and welfare of the most marginalized, often they do not. So it was likely that any initiative that we supported might conflict with existing power structures and accountabilities - either formal or informal. Accordingly, if not well conceived, our interventions could undermine our end goal by potentially strengthening accountability relationships that were not broadly supportive of equitable health services.

Second, as already noted, recent literature has underscored the difficulties of institutionalizing new measures to promote accountability within health systems. These include logistical challenges involved in taking to scale a complex package of interventions that needs tailoring to different communities; ensuring financial support for social accountability programs beyond the end of donor-supported projects; sustaining community engagement and enthusiasm given multiple other demands upon community time; and ensuring the political acceptability of an approach that is ultimately seeking to engender a more equitable balance in power between the disadvantaged and the privileged. This latter point about the political acceptability of social accountability measures is key. Without support from political elites, it will likely be challenging to acquire the necessary resources (financial and human) to institutionalize a social accountability initiative within government. While multiple efforts have been made to scale up social accountability measures outside of government, these (with a few exceptions) also struggle to sustain themselves due to a lack of access to a steady source of support [6]. Further, it has been convincingly argued that without reinforcement and support from higher levels of government, social accountability schemes are unlikely to work well [14].

Third, and finally, FHS had a central focus on equity. A question that we were very concerned about centered on the degree to which social accountability approaches promoted equity and inclusiveness, versus giving voice to a stratum of society who were already relatively privileged. There is much that is still poorly understood about this question. For example, while one study in India that explicitly sought to assess involvement of more marginalized populations in social accountability initiatives concluded that bureaucratic and language barriers typically worked to exclude the most marginal populations, the authors also noted that it was nonetheless possible that these instruments worked in the interest of these marginalized populations, even if they did not directly participate [15]. Another paper, reviewing the experience of the large non-governmental organization CARE, noted that while social accountability initiatives strive for diverse participation, evidence on inclusiveness and equity is thin [16].

## The FHS approach to social accountability

The work reported in this supplement was conducted as part of a 2 year pilot project for community scorecards in Bangladesh and Uganda. At the time of planning this work, we hoped that the communities would be able to sustain the interventions on their own or that there would be follow-on funding that would allow the teams in these sites to scale up the interventions developed through this formative work, and evaluate their impact on services and equity. Sadly, this has not been the case, however from the start we approached the design, implementation and assessment of these initiatives as if their primary purpose was to inform the design of a scalable social accountability initiative that could fundamentally shift the dynamics of health system accountability in favor of poor and marginalized people. To this end, there were a number of defining characteristics of our approach.

From the start of the project, we had a purposeful awareness to both formal and informal relationships in the accountability ecosystems in both countries. As part of our planning processes, and prior to any intervention work in the two countries, we conducted a mapping of accountabilities using a tool adapted from Brinkerhoff that described the capacity that different actors had to demand information of each other, impose sanctions on each other, or supply information [17]. In both countries these mappings emphasized the existing complex networks of accountabilities, and also prompted long discussions among the research teams seeking to parse out formal accountabilities from informal norms. This process also helped to identify which stakeholders it would be critical to bring to the table in order to ensure buy in for the proposed initiative [18].

Monitoring, evaluation and learning were central to our efforts to implement the community scorecards. In particular, we were eager to understand how and why our intervention was working or not. Wild and Harris [19] suggest multiple different channels through which social accountability approaches might function including strengthening citizens demand, increasing resourcing, improving information flows, creating greater top down pressure for performance improvement, and promoting collective action on the part of communities, or potentially across communities and providers. We realized that understanding which of these mechanisms was operational, was important. Such information would help us to adapt or trim back components of the intervention that did not appear to be relevant, or strengthen those that were weak. Early on in the project, the teams in both Bangladesh and Uganda developed theories of change that spelled out how their planned interventions might impact the relationships between service providers and communities, as well as the broader accountability ecosystem in each setting. The paper by Kiracho et al. in this volume [20], further considers the evidence from Uganda on which of these mechanisms were important in that particular context.

Alongside the routine monitoring and evaluation that the project had planned, we also carried out a more in-depth process documentation. This had a dual purpose. We wanted to understand what it took to launch these community scorecards, and therefore the project teams required detailed records of the type and nature of support provided and activities undertaken, so that over time, the program structures could be reviewed, refined and streamlined. This detailed documentation was a critical input for the costing study reported in this supplement by Ssebagareka et al. [21]. The project documentation also served a second purpose to understand the implications for equity and inclusiveness. Through these records, we kept careful track of who was participating in the community scorecard processes (such as consultative and interface meetings) and by extension, who was not doing so. Apolot et al. (this supplement [22]) also drew upon this documentation to consider participation in community scorecard services, and supplemented it with interview data, so as to consider how the scorecards affected one particularly marginalized group in Uganda, namely adolescent mothers. Mahmood et al. (this supplement [23]) used this data along with other interview data to identify the level of participation among female and adolescent facility management group members in the scorecard process and its impact on health service use among these marginalized groups.

As the teams developed country-specific drafts of theories of change, we also pondered questions related to the institutionalization and sustainability of our initiatives. We recognized that full commitment to institutionalizing these programs would affect the design decisions that we made from the start. However, it quickly became apparent that this commitment to institutionalization often implied compromises. Both country teams identified implementation options that would align their initiatives with current government priorities. In Uganda, the government was seeking to roll out a scorecard for maternal, neonatal and child health services. However, the model the government was supporting did not involve community engagement. The FHS team engaged with the Ministry of Health to explore their openness to piloting an alternative version of the proposed scorecard, that did engage communities in its construction and also might include measures related to community obligations (such as early care seeking for antenatal care). In Bangladesh, since 1996 the government had sought to scale up a series of community clinics across rural parts of the country to provide accessible and community-oriented primary care services [24]. However, these facilities remained under-utilized, and the community committees that were intended to oversee their operations were barely functional. This created an important opportunity to align the FHS intervention with existing systems and recognized policy needs.

In both cases, closely aligning the FHS interventions with government policy came with potential risks. Potentially it could undermine the perceived independence of the initiative, sending a signal to other stakeholders that this was business as usual, and perhaps deterring some sections of society from participating. We were also apprehensive about whether government representatives at different levels of the health care system would take this initiative seriously, or view it as an infringement upon their autonomy to direct health programs as they wished. Nonetheless, and as described further in papers in this volume by Mahmood et al. [23] and Kiracho et al. [20], we concluded that based on our project goals, the nature of our networks in each country, and an over-riding concern with sustainability, it made sense to pursue a strategy that aligned closely with government policy directions from the start. Engagement with civil society was further constrained by their very minimal presence at the local level. In Uganda, for example, we were able to identify only one small civil society group that was already overburdened with its primary responsibilities. As we refined our theories of change, we sought to embed constructs related to institutionalization, scale and sustainability within them, as a means to keep ourselves focused on these important objectives throughout project implementation.

## Implications of our work

We will not foreshadow the findings of the individual papers in the supplement here*.* Instead we reflect on some of the findings emerging, and how these relate to our initial hopes - and concerns - about social accountability approaches.

We must recognize that our research teams played pivotal roles in the implementation of the scorecards in both countries, an approach which highlights how resource-intensive research-driven social accountability endeavors, such as ours, can be. The paper by Ssebagareka et al. [21] provides a clear financial account of the costs involved in this project, and how they might have been modified if more responsibilities were born by health and other non-health staff on the ground, or if the community engagement process had been less intensive. In Bangladesh, where there was no comparable financial analysis, it is still clear that considerable resources were expended. Within the space of 10 months and three cycles of scorecards the study team facilitated 44 meetings involving 643 participants [25]. Would it have been possible to achieve the same outcomes with less effort and fewer resources? Our teams have reflected on this question. While costs would likely lower over time as the processes involved in CSC become routinized, there was general consensus that high start-up costs, particularly for capacity building and community sensitization, were necessary in order to be able to implement the intervention effectively. Further, despite the best efforts of FHS country teams to build capacity, by the end of the project, neither the Uganda or Bangladesh teams felt fully confident that the facilitators they had trained locally would be able to continue to lead the processes without ongoing support from FHS. This was for a number of reasons, including high turnover among the facilitators, the need for refresher trainings, and the simple fact that such facilitation takes time, and there were few local actors who had relevant facilitation skills to start with. Further, in neither of the contexts where we were working was there a robust civil society, and so it was difficult to identify other actors who could guide this process. While training and retaining skilled facilitators was challenging, teams in both Bangladesh and Uganda recognized the critical importance of facilitations skills to guide sometimes difficult discussions between communities, health staff and local leaders.

Our data on the inclusivity of these schemes is partial, but the paper by Apolot et al. [22] underscores the challenges of ensuring that community scorecard initiatives such as this can respond effectively to needs and preferences that lie outside of the mainstream. While we believe that the structures and procedures followed by the teams helped to ensure that poorer households within the community at least had a seat at the table, the experiences of vulnerable community sub-groups, such as adolescents in Uganda, were really not addressed within the scorecard process, and one can imagine that other marginalized groups, particularly those who face stigma (for example, men who have sex with men, or people living with disability) would also be unlikely to participate in such exercises. Recognizing the likely limitations of community scorecard processes, and finding other, more private ways to consult with certain stigmatized marginalized groups to better understand their needs and preferences will be important.

While the FHS team had hoped that it would have a longer period of time to embed these initiatives within local systems and structures, without follow-up project support after the 2 year pilot period, it has been challenging to maintain these initiatives. Further, as reported by Mahmood et al. [23], our initial concerns about the likely degree of commitment local leaders would have to social accountability initiatives was not unfounded. Nonetheless, despite the lack of external follow-on funding the approach we adopted has led to some promising local developments. In Bangladesh the scorecard initiative has resulted in some lasting impacts within the facilities where it was initiated such as observance of posted service hours, improved and regular communication between facility management, community and the local government representatives, continued active participation of facility committee members in management of the facilities, and sharing of regular updates on clinic activities at district level meetings. At the national level, the concerned office at the Ministry of Health and Family Welfare now consults with the FHS Bangladesh team in designing interventions to improve service provision at the community clinics. The work in Uganda showed that the key players in maternal and newborn health were indeed willing to engage with communities in light of the high maternal and neonatal mortality and morbidity. In the district where the project was implemented, both health and non-health leaders at the district and local level have continued to work closely together, and to actively engage health workers in dialogue when negative feedback is received about services.

## Conclusion

The FHS experience with social accountability initiatives reinforces much of what is already in the literature: such initiatives are challenging to take to scale; may not include the marginalized or most vulnerable; are resource intensive to initiate; and may be resisted by more powerful local stakeholders. Our experience described in the papers in this supplement, however, also resonate with the optimism in the literature about the power and potential of social accountabilities to change quite fundamentally the relations between community leaders, health care providers and communities in ways designed to strengthen health service and build social capital within the community. Without better accountability at the local level, many other investments in health are likely wasted. Frequently, the structures to support social accountability are in place, but are badly neglected. For these reasons, it seems as if the fight to improve social accountability within the health system is one worth fighting, but we need to be prepared for a long and drawn out battle. Finally, FHS experience underscores the need for both researchers and practitioners working in social accountability to test approaches for customizing their interventions to what is most feasible and effective locally. While we should continue to build an evidence base on the effectiveness of social accountability interventions, the questions focused on implementation and scale in different specific contexts remain important.

## Data Availability

Not applicable – this paper is an editorial describing the context and content of other papers included in this special issue. It does not involve primary data.
